# From the Editor’s Desk

**Published:** 2010-08

## Advance publications

The office of the *Cardiovascular Journal of Africa* has recently experienced a dramatic increase in the number of articles submitted to this journal. The articles emanate from authors in many diverse countries. As a consequence of the volume of articles, we are experiencing delays in the length of time to publish them.

To obviate this, we have now made available an additional option for authors to advance publish their articles online. We must charge for this option as it involves additional expense. Local authors in Africa will pay R1 000 and those overseas R2 500. This amount could be budgeted for in the research grant if one knows beforehand that the article needs to be advance published.

Authors will now have the immediate benefit of having their article appear online on PubMed, but will have to wait as usual for the article to appear in the printed journal. We trust that this will be of help in getting your important research to the immediate attention of other interested researchers and readers.

**Fig. 1.**
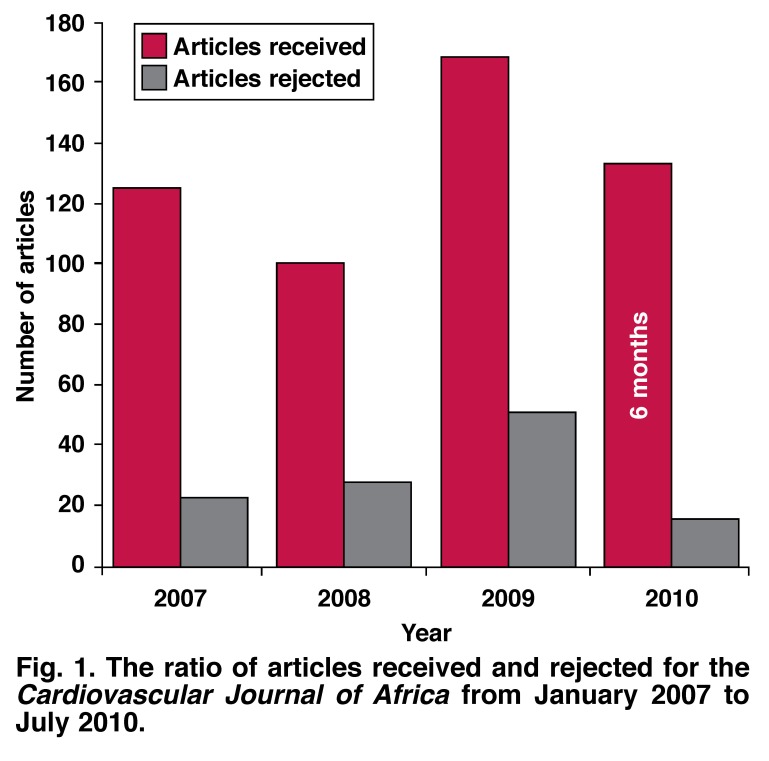
The ratio of articles received and rejected for the *Cardiovascular Journal of Africa* from January 2007 to July 2010.

Please contact Elsabé Burmeister at elsabe@cvja.co.za for more information. Fig. 1.

